# Biogenic Tooth-Integrated Fixed Functional Space Maintainer for Pediatric Use: A Novel Approach

**DOI:** 10.7759/cureus.63621

**Published:** 2024-07-01

**Authors:** Aakriti Chandra, Sakshi P Kabra, Ramakrishna Yeluri, Nilima R Thosar, Monika Khubchandani, Neha Pankey, Meenal S Pande, Garima Yeluri

**Affiliations:** 1 Pediatric and Preventive Dentistry, Sharad Pawar Dental College and Hospital, Datta Meghe Institute of Higher Education and Research, Wardha, IND; 2 Pediatric and Preventive Dentistry, Datta Meghe Institute of Medical Sciences, Wardha, IND; 3 Pedodontics and Preventive Dentistry, Sharad Pawar Dental College and Hospital, Datta Meghe Institute of Higher Education and Research, Wardha, IND; 4 Pediatric Dentistry, Sharad Pawar Dental College and Hospital, Datta Meghe Institute of Higher Education and Research, Wardha, IND; 5 Oral Medicine and Radiology, Consultant Oral Physician and Radiologist, Wardha, IND

**Keywords:** fixed functional space maintainer, natural teeth, avulsion, primary anterior teeth, pediatric dental trauma

## Abstract

Preschoolers frequently experience traumatic dental injuries, particularly during their two to four years of life. The majority of these injuries result in tooth avulsion because of the alveolar bone resiliency around the primary teeth. This study explains an instance of damage sustained during play that resulted in an early knockout of the primary incisor. Hence, a biogenic tooth-integrated space maintainer was created using the natural crown of the traumatized tooth, and the child was asked to come for a regular follow-up. This treatment may be viewed as a great alternative for natural aesthetic rehabilitation as it promotes speech development, improves oral cleanliness, restores aesthetics and masticatory function, and inhibits the development of aberrant tongue habits and malocclusions.

## Introduction

Traumatic dental injuries occur most commonly in the primary dentition of children and young adults, which accounts for about 22.7% of all dental injuries [1]. Early loss of the primary anterior teeth is mainly caused by injury that can happen because of knocking out [2], elimination after getting worse due to poor prognosis [3], complications that occur long after the accident, or early shedding process [4]. Avulsion happens commonly among children aged between two and four years [5], as young children try to learn motor movement followed by children aged between six and 10 years, which is the time period for the eruption of permanent incisors [6]. It hits boys at a higher number than girls at a ratio of 1.2:1.5 [7]. The maxillary primary central incisor is mainly affected than any other teeth followed by maxillary lateral incisors and mandibular central incisors [8-10].

Avulsion of a primary incisor is frequently accompanied by luxation injuries to adjacent teeth [11], facial bone fractures [12], and the surrounding gingival tissue as well as lip lacerations [13]. The three possible treatments for an accident where the upper front teeth are completely out of the socket include no treatment (i.e. not putting them back in the mouth) [14], replacing the missing ones with prosthetic teeth [15, 16], or reimplantation of the avulsed tooth [11].

Whenever exarticulation of primary teeth occurs, the alveolar socket is observed to be empty, which can be confirmed by periapical radiographs. It is considered one of the rarest luxation injuries [6]. Given that such injuries can occur anywhere, a child could be with or without parents, in school, or in kindergarten; thus teachers in kindergarten need to be able to handle them. They must remain composed and should not panic, identify the nature of the child’s injury if any at all, assess if the injury is life-threatening, and look for signs if he or she is suffering from amnesia, loss of consciousness, or vomiting.

According to the International Association of Dental Traumatology (IADT) [17], it is not advisable to replant deciduous teeth because several problems may arise in the long run [18]. Some researchers also say that reinserting deciduous teeth might be an unreasonable idea since it can result in infections in the jawbone where the permanent tooth is located or even the tooth [19].

## Case presentation

A four-year-old female patient accompanied by her parents presented to the outpatient department of Paediatric and Preventive Dentistry, Sharad Pawar Dental College and Hospital (SPDC) with complaints of a complete knockout of the upper front tooth from the socket due to injury in the upper jaw while running in the home yard in the morning.

Clinical examination

**Figure 1:**
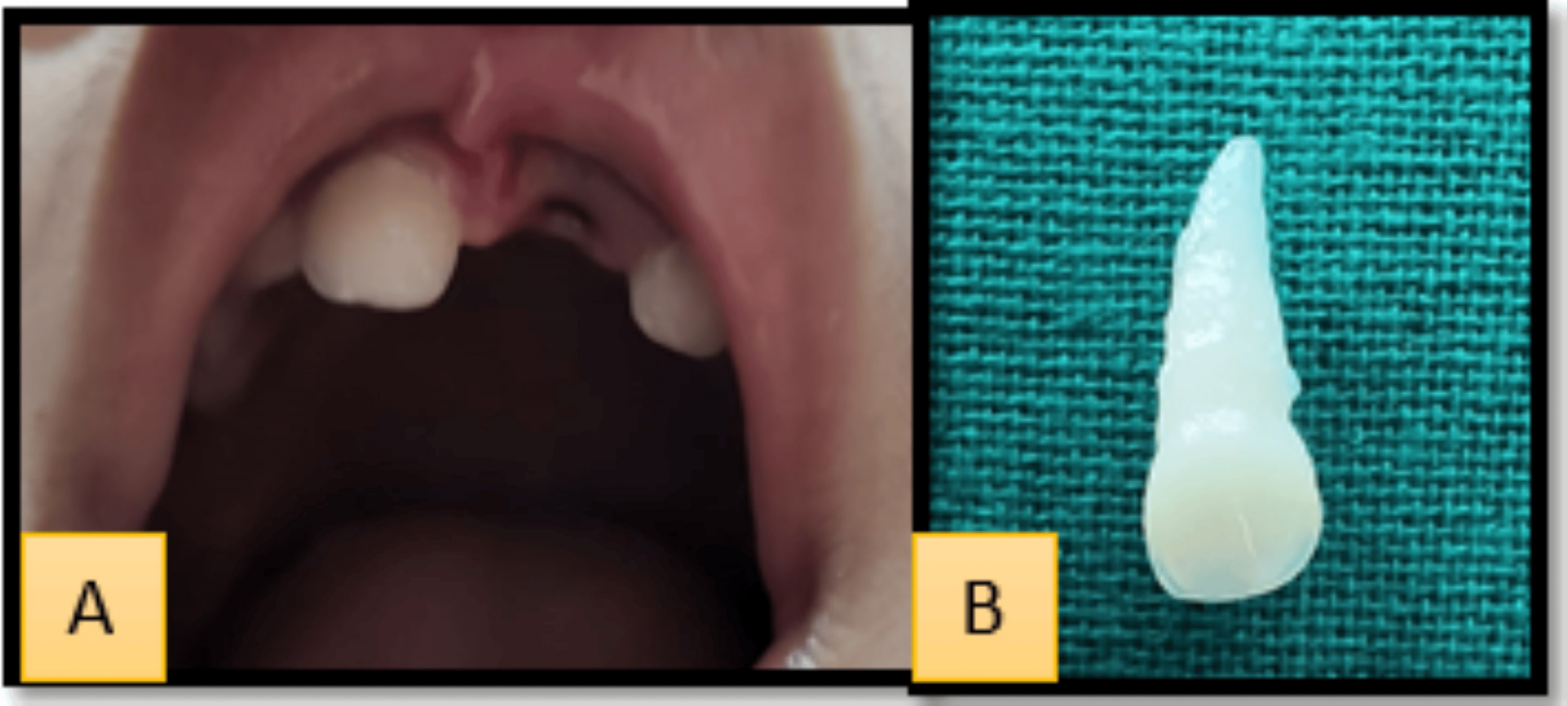
A. Avulsion of 61 due to trauma. B. Avulsed tooth

After reviewing the traumatic history and conducting a clinical intraoral examination, a conclusive diagnosis of a complete traumatic avulsion of tooth 61 was made (Figure 1). The tooth was brought in a glass bottle filled with regular tap water. Also, the extra-oral examination revealed no significant findings. As the patient behavior rating was 1 (definitely negative) according to the Frankl behavior rating scale, a clear radiograph could not be obtained. The clinical examination of the avulsed tooth revealed no fracture of the tooth root or any attached bone fragment indicating no fracture of the alveolar bone.

Proper procedural consent was obtained from the parents followed by the treatment performed for the patient. Since the reimplantation of a primary tooth is contraindicated as per IADT guidelines and considering the patient's age, it was planned to replace the empty socket esthetically with a biogenic fixed functional space maintainer utilizing the patient's own natural tooth.

First appointment

**Figure 2:**
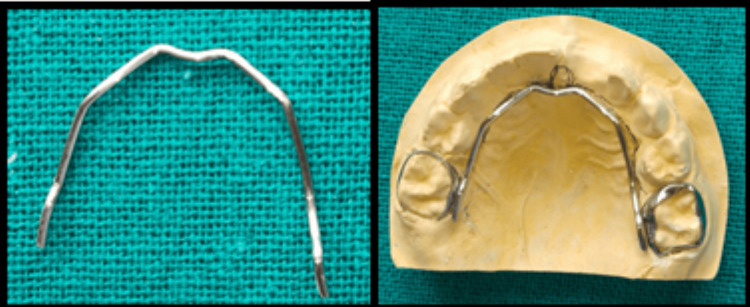
Stainless steel framework

Before the construction, the mesiodistal and buccolingual width of the primary second maxillary molars were measured and accordingly, prefabricated bands were selected for the same. After the band selection, tray selection was done for the upper arch, which was found to be U0. Using the fast-set alginate impression material, the impression of maxillary arch was made and immediately the cast was poured using type III gypsum product (dental stone) with the appropriate water:stone ratio. On the maxillary cast, after marking the area, a stainless steel framework was constructed, extending from tooth 55 on one side to tooth 65 on the other, resembling a Nance palatal arch, relieving the incisive papilla region (Figure 2).

Fabrication of biogenic space maintainer

**Figure 3:**
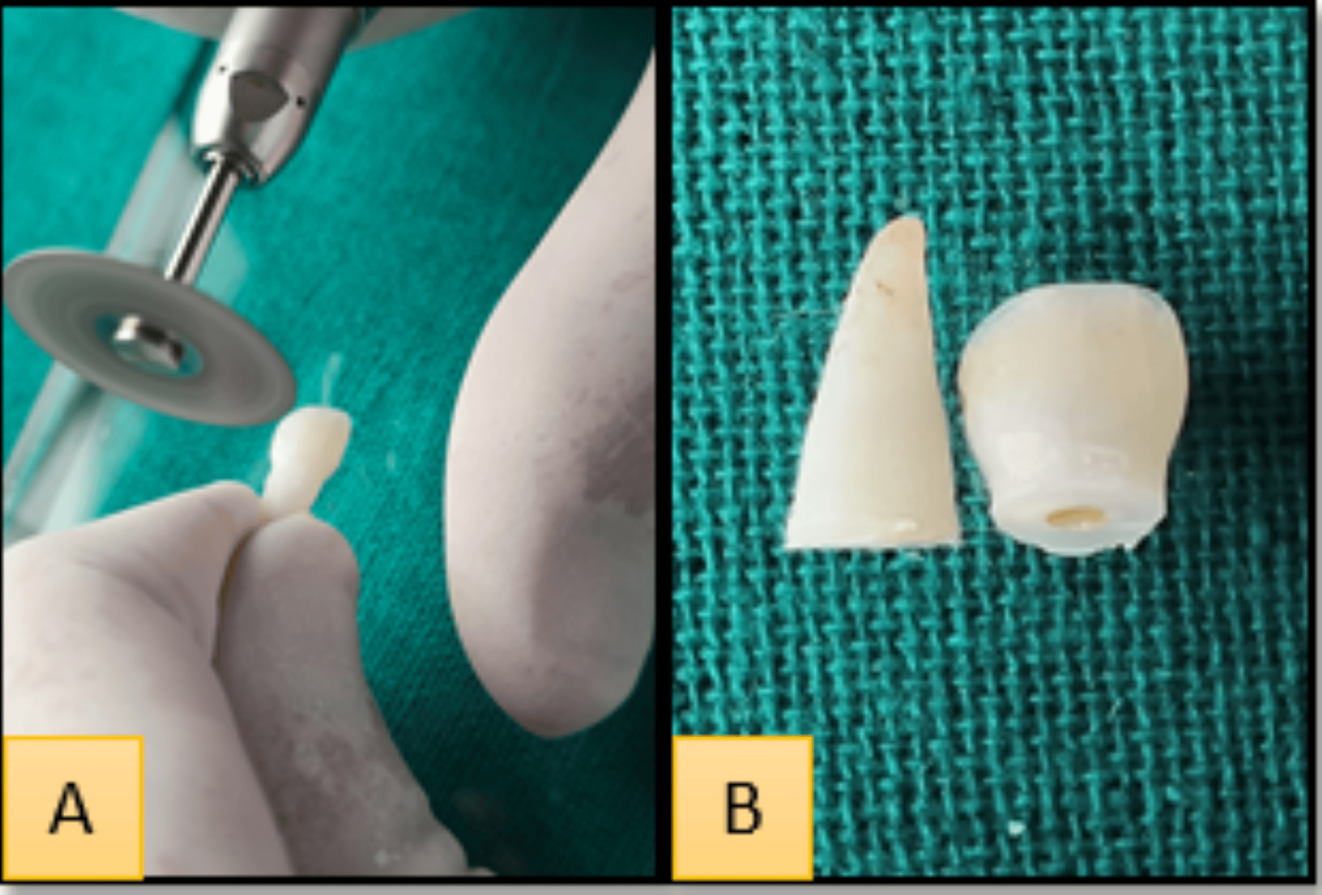
A. Sectioning of the tooth using diamond disc bur. B. Sectioned tooth (coronal and radicular halves)

**Figure 4:**
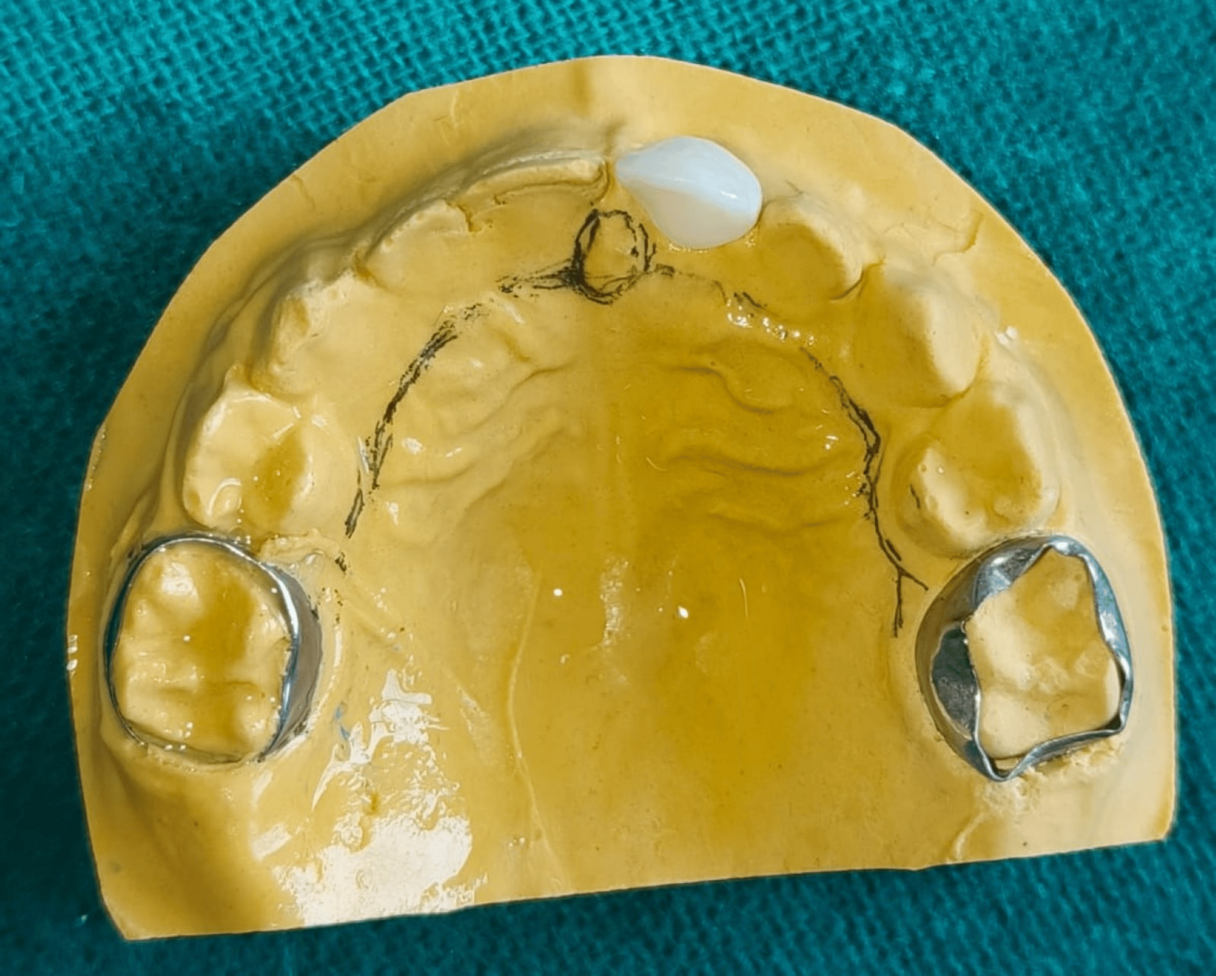
The trimmed natural tooth adjusted according to the flange of the alveolar bone

The exarticulated tooth kept in water was then taken out and, under all aseptic conditions, it was split horizontally into two halves (coronal and radicular) using a diamond disc bur (Figure 3). The coronal portion of the tooth was made even from cervical end and a ditch was given, creating access to the pulp chamber. The tooth was made free of all the remaining pulpal tissue and thorough irrigation was done using a saline solution. The pulp chamber was then etched using etching gel for about one minute followed by the application of bonding agent using an applicator tip. The pulp chamber was filled with flowable composite, thus sealing the tooth completely. This natural tooth was then trimmed and adjusted according to the flange of the alveolar bone (Figure 4).

**Figure 5:**
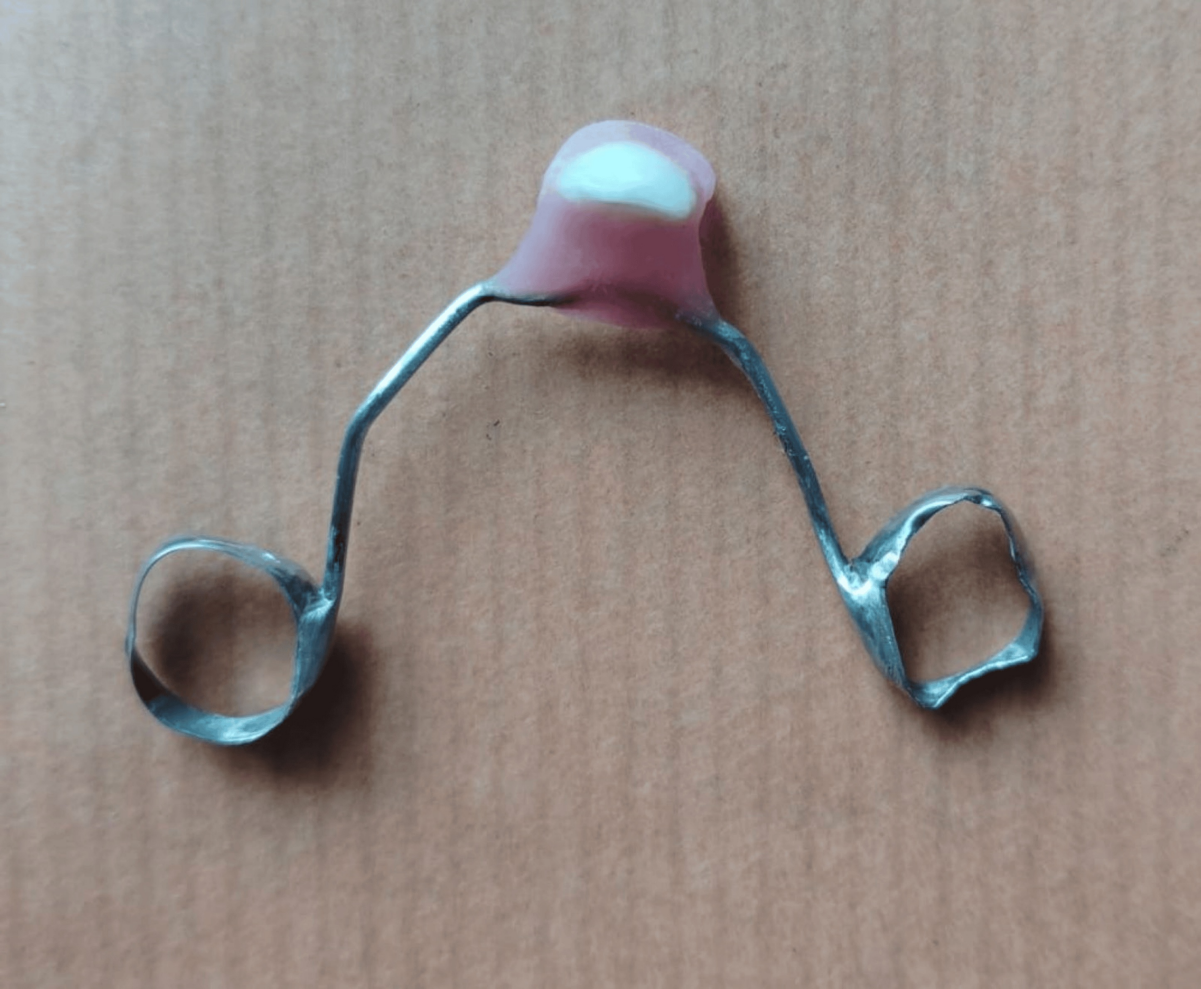
Fabricated space maintainer

The wire framework was then soldered to the bands on the chosen abutments. An acrylic extension was applied from the palatal area to the labial vestibule over the wire. The processed biogenic natural tooth was positioned directly over the alveolar crest on the acrylic extension (Figure 5).

Second appointment

**Figure 6:**
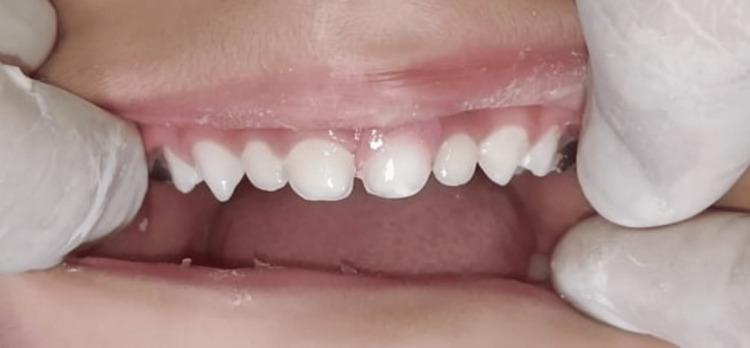
Post-operative image after cementation of the space maintainer in the patient's oral cavity

**Figure 7:**
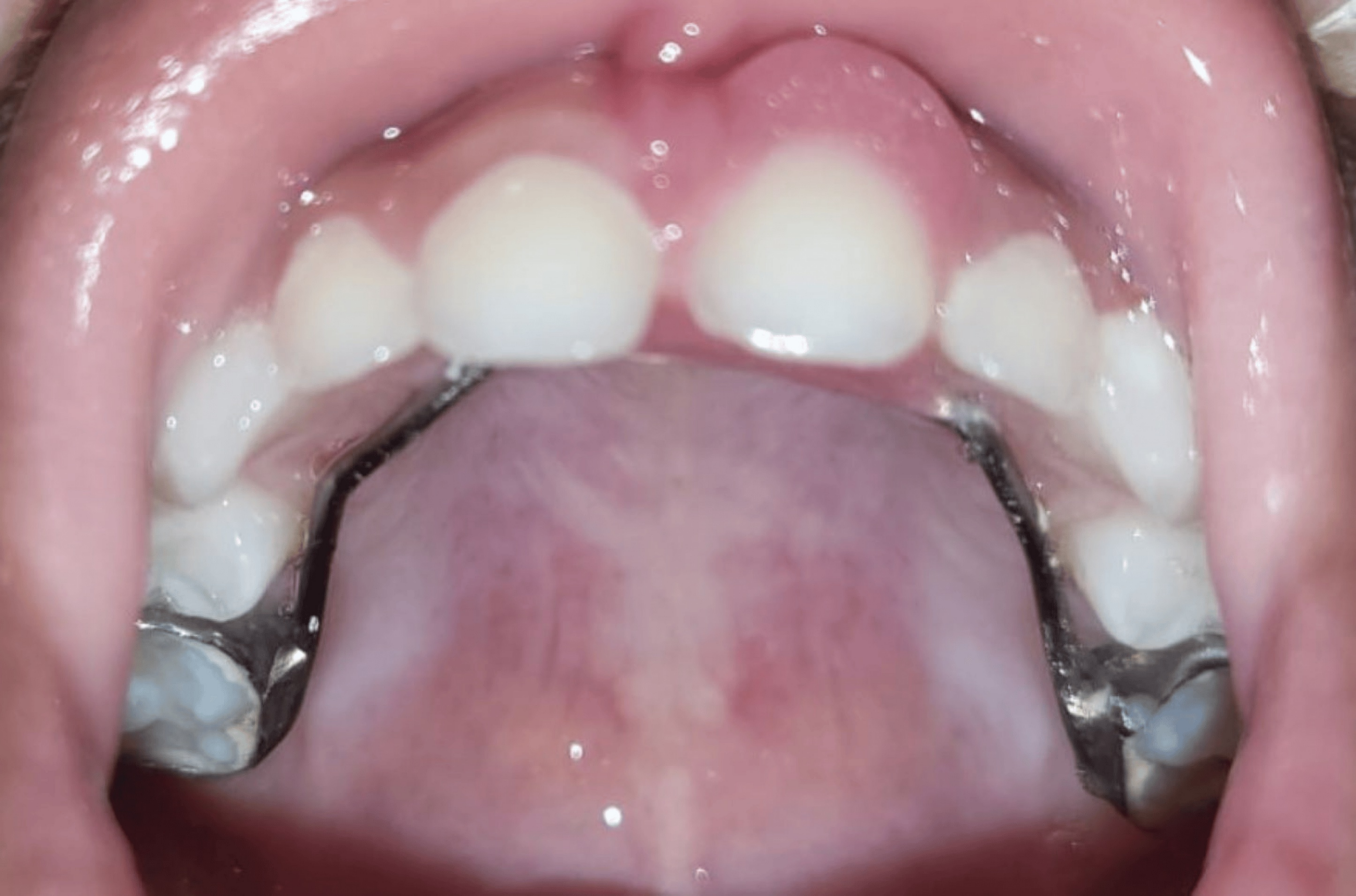
Post-operative image after one-week follow-up

Occlusion was then checked and adjusted both on the cast and in the patient's mouth. After the required trimming and polishing, the appliance was fitted and cemented to the abutments using Type I glass-ionomer cement (Figure 6). Proper oral hygiene maintenance instructions were provided to the child and her parents. They were also advised to visit the outpatient department for regular dental check-ups. The post-operative period was uneventful and the child readily adjusted to the biogenic space maintainer (Figure 7). 

## Discussion

The flexibility of the alveolar bone surrounding the primary teeth causes most preschoolers to have dislocated dental problems such as avulsion of teeth and luxation injuries [20]. Some of the effects of trauma on primary teeth will be discoloration, dead pulp (pulp necrosis), filling up of the pulp canal over time (pulp canal obliteration), gum moving further inwards toward the middle line of the teeth (gingival retraction), losing a baby tooth (displacement of a primary teeth), abnormal shortening of tooth roots (pathological resorption), changes in physiological root resorption, and eventual early exfoliation of that particular tooth [21]. According to IADT, reimplantation is contraindicated in the primary tooth [17, 18]. Hence, in this case, it was planned to replace the empty socket with the patient's own natural tooth.

At this point, the main problem needs to be addressed. It concerns the aesthetic issues that assume importance at the preschool stage because the children can discern these standards from their friends and peers and thus have preferences [22]. The patient’s self-image and esteem were required for the decision to construct an aesthetic space maintainer.

Since many sounds are produced through the tongue touching the lingual side of the upper incisors and if these teeth are missing, there can be wrong speech compensations [23]. This is not only for beauty reasons but because masticatory function would be affected, speech development would interfere with the establishment of tongue habits, and there are also worries over space maintenance [24].

Natural teeth have several advantages related to dental rehabilitation and function restoration. These include better aesthetics, physiological wear, and smoother surfaces [25], which help to reduce biofilm retention and promote easier oral hygiene maintenance. The ability to deal with tooth loss more readily is one of the main advantages of keeping the patient's native crown [26]. It also provides the optimum choices concerning size, alignment, color, and form [27]. The benefits of using cosmetic space maintainers in conjunction with natural teeth were a factor in the effective treatment and patient and family satisfaction.

The tooth should not remain outside the mouth for long periods as they tend to lose moisture. Teeth that remain outside the oral environment need careful management; this includes storage under moist conditions for a day and sectioning off their clinical crown through a slow-speed handpiece.

According to Orsi et al., a removable space maintainer is the preferred option when one or more than one primary teeth are lost as it does not interfere with the development of teeth and the dental arch [28]. In contrast, fixed appliances require pre-contoured stainless steel or orthodontic bands, which can extend treatment time and pose challenges with uncooperative children [29]. In a case report, Kupietzky also described a fixed appliance similar to a Nance holding arch, attached to the primary molars using prefabricated stainless steel bands [15].

Furthermore, a study on the prosthetic restoration of avulsed primary teeth on detachable aesthetic space maintainers utilizing natural primary teeth was described by a group of authors from Brazil, similar to the one in our study. Within 12 months, this approach was assessed, which showed positive effects in children with acceptable aesthetics, normal speech development possibilities, good masticatory function, and easy oral hygiene maintenance [30].

The main drawback of these fixed appliances is the accumulation of food residue and plaque formation [31]. Therefore, parents must be trained thoroughly to supervise their child's oral hygiene practices.

## Conclusions

A novel approach to replacing a lost anterior tooth with natural teeth is suggested as discussed in this article. Compared to a removable appliance, it offers greater comfort to the younger patient. With many benefits, a natural tooth crown placed in an aesthetically pleasing space maintainer can be a great rehabilitation choice for anterior primary tooth loss. In addition to being simple to make and install, it improves oral cleanliness, helps to avoid aberrant tongue habits and malocclusions, restores aesthetics and masticatory function, and supports speech development. Additionally, the patient's parents received oral hygiene advice to follow at home.
